# The 100 most influential publications in paracetamol poisoning treatment: a bibliometric analysis of human studies

**DOI:** 10.1186/s40064-016-3240-z

**Published:** 2016-09-13

**Authors:** Sa’ed H. Zyoud, W. Stephen Waring, Samah W. Al-Jabi, Waleed M. Sweileh, Rahmat Awang

**Affiliations:** 1 Poison Control and Drug Information Center (PCDIC), College of Medicine and Health Sciences, An-Najah National University, Nablus, 44839 Palestine; 2 Department of Clinical and Community Pharmacy, College of Medicine and Health Sciences, An-Najah National University, Nablus, 44839 Palestine; 3 WHO Collaborating Centre for Drug Information, National Poison Centre, Universiti Sains Malaysia (USM), 11800 Pulau Pinang, Penang Malaysia; 4 Acute Medical Unit, York Teaching Hospitals NHS Foundation Trust, Wigginton Road, York, YO31 8HE UK; 5 Department of Pharmacology and Toxicology, College of Medicine and Health Sciences, An-Najah National University, Nablus, 44839 Palestine

**Keywords:** Paracetamol, Acetaminophen, *N*-acetylcysteine, Bibliometric, Citations, Scopus, Poisoning

## Abstract

**Background:**

Analysis of the most influential publications within paracetamol poisoning treatment can be helpful in recognizing main and novel treatment issues within the field of toxicology. The current study was performed to recognize and describe the most highly cited articles related to paracetamol poisoning treatment.

**Methods:**

The 100 most highly cited articles in paracetamol poisoning treatment were identified from the Scopus database in November 2015. All eligible articles were read for basic information, including total number of citations, average citations per year, authors’ names, journal name, impact factors, document types and countries of authors of publications.

**Results:**

The median number of citations was 75 (interquartile range 56–137). These publications were published between 1974 and 2013. The average number of years since publication was 17.6 years, and 45 of the publications were from the 2000s. A significant, modest positive correlation was found between years since publication and the number of citations among the top 100 cited articles (r = 0.316; p = 0.001). A total of 55 journals published these 100 most cited articles. Nine documents were published in *Clinical Toxicology*, whereas eight documents were published in *Annals of Emergency Medicine*. Citations per year since publication for the top 100 most-cited articles ranged from 1.5 to 42.6 and had a mean of 8.5 citations per year and a median of 5.9 with an interquartile range of 3.75–10.35. In relation to the origin of the research publications, they were from 8 countries. The USA had the largest number of articles, 47, followed by the UK and Australia with 38 and nine articles respectively.

**Conclusions:**

This study is the first bibliometric assessment of the top 100 cited articles in toxicology literature. Interest in paracetamol poisoning as a serious clinical problem continues to grow. Research published in high-impact journals and from high income countries is most likely to be cited in published paracetamol research.

## Background

*N*-acetylcysteine (NAC) is a well-established antidote for a paracetamol (acetaminophen) overdose and is highly effective in minimising liver injury if administered promptly. However, NAC is associated with a number of adverse effects, including nausea, vomiting and non-immunoglobulin E anaphylactic (anaphylactoid) reactions (Zyoud et al. 2010c). A number of methods have been applied in clinical practice for risk stratification, such that NAC is administered to patients thought to be at significant risk of paracetamol-induced liver injury. Most prominently, the extent of paracetamol exposure may be estimated from a measured serum concentration at a known interval after ingestion. However, there are difficulties in relying on patient reporting, and errors may occur concerning the reported drug name, dose and timing of ingestion (Zyoud et al. 2012; Hewett et al. 2013; Rutter et al. 2013). A history of acute or chronic alcohol ingestion and biochemical tests of malnutrition have been explored as means of detecting an increased susceptibility to paracetamol liver toxicity; however, none of these are sufficiently reliable for routine clinical application (Waring et al. 2008a, b, d; Zyoud et al. 2011).

The original NAC infusion regimen described by Prescott in the late 1970s gives rise to very high initial blood concentrations, which are associated with the development of adverse effects; a number of alternative regimens have been described, which incorporate a slower initial rate of infusion (Prescott et al. 1977; Zyoud et al. 2010a, b; Waring 2012). Novel NAC infusion regimens are associated with a lower rate of occurrence of adverse effects, but too few data are currently available for a comparison of efficacy in preventing paracetamol-induced liver injury (Chiew et al. 2016). Nonetheless, the regulatory authorities in the UK made substantial changes to the NAC Marketing Authorisation in September 2012, incorporating a slower delivery of the initial loading dose of 150 mg per kg, extended from 15 to 60 min. At the same time, amendments were made to the criteria for NAC so that treatment should be considered for any patient that ingests more paracetamol than 75 mg per kg of body weight or has a measured paracetamol concentration higher than the 100-line nomogram that was formerly used for “high risk” patients only. Other countries have followed suit and incorporated these amendments into local policy. At present, few data exist concerning the impact on rates of occurrence of liver injury or adverse acetylcysteine effects. Early reports from the UK indicate that, since September 2012, there have been increased numbers of patients receiving acetylcysteine, increased hospitalisation and a substantial financial burden (Thompson et al. 2013; Bateman et al. 2014a).

Other novel therapeutic approaches have been examined in patients receiving an intravenous NAC antidote after paracetamol poisoning. For example, co-administration of cimetidine has been found to have no significant effect on patient outcome (Ebrahimi et al. 2015). Co-administration of ondansetron is highly effective in reducing the adverse effects associated with NAC administration, and this might be considered for routine administration in patients at high risk of anaphylactoid reactions, for example those with a history of asthma or previous adverse effects after NAC (Bateman et al. 2014b).

While numerous bibliometric reports have been performed to investigate factors related to research output in the toxicology field (Zyoud et al. 2010b, 2014a, 2015a, b, c, d), to our knowledge, no study has attempted to evaluate the most influential publications within a particular subspecialty in toxicology. The main aim of our study was to identify the 100 most frequently cited articles related to paracetamol poisoning treatment. Therefore, a bibliometric evaluation of scientific literature in a particular field may be used to recognize the impact of influential scholarly work, authors, subjects, countries, etc. The total number of citations that a published article has achieved indicates the importance that published article has on that area of practice. Analysis of the most influential publications within paracetamol poisoning treatment can be helpful in recognizing main and novel treatment issues within the field of toxicology.

## Methods

### Data acquisition

In November 2015, we used the Scopus database to retrieve the most frequently cited articles on paracetamol poisoning treatment. Scopus is the largest electronic scientific database. It is larger than either MEDLINE or Web of Science and is more accurate than Google Scholar. Furthermore, Scopus is accessible and allows researchers to obtain information and do analysis that might not be readily achievable in MEDLINE or Google Scholar.

The search terms used to retrieve articles related to paracetamol poisoning treatment were elected from previous bibliometric studies related to paracetamol (Robert et al. 2009; Zyoud et al. 2015a, d). The articles entitled with the terms “acetaminophen” or “paracetamol” or “acetamidophenol” or “hydroxyacetanilide” or “tylenol” or “*n*-acetyl-*p*-aminophenol” or “panadol” or “APAP” or “acephen” were included in the research. Then, the following keywords were entered as terms in the article title or abstract: “poison*” or “overdose*” or “*toxic*”. All the previous terms were followed by “treat*” or “antidot*” or “detoxification” or “intervention” or “manag*”. To include all possible publications related to paracetamol treatment, we included the following strategy in addition to the previous one. The following keywords, “NAC” or “acetylcysteine” or “*N*-acetyl-l-cysteine”, were entered as terms in the article title or abstract and followed by the following terms: “acetaminophen” or “paracetamol” or “acetamidophenol” or “hydroxyacetanilide” or “tylenol” or “*n*-acetyl-*p*-aminophenol” or “panadol” or “APAP” or “acephen”. The Scopus search was conducted by applying the previous strategies of search for all previous years. No limits were placed on the time period for this search. Furthermore, all documents related to paracetamol, with no language restriction, were searched in Scopus database. Wildcard characters were used to include variations of a word by using an asterisk (*) to make our search strategy simpler. For example, in the Scopus search engine, when we entered “*toxic*”, it offered a wildcard character capability, and we got results for toxic, toxicity, toxicant, toxicities, intoxication—briefly, any possible word that might include the five letters (i.e. toxic).

### Data analysis

We sorted the extracted results from the largest number of citations to the lowest. The results were then evaluated by two independent researchers to extract only the top 100 cited articles related to paracetamol poisoning treatment in human research subjects. Total numbers of citations, average citations per year, authors’ names, journal name, impact factors (IF), document types, countries of publication, and number of authors for only the top 100 cited articles were noted. The impact factor was obtained from 2014 Journal Citation Reports^®^ (Thomson Reuters, New York, NY, USA) (Thomson Reuters 2015) for all journals that published the top 100 cited articles. This is useful in clarifying the significance of absolute citation frequencies. This search used the electronic version of this database by ranking the top 100 cited publications using the standard competition ranking (SCR). It is possible that some articles were cited more frequently than others because of the long time elapsed since their publication. Therefore, a citation index was calculated for each article to avoid the bias created by the time elapsed since publication. Citation index is calculated as the average number of citations divided by number of years elapsed since the article was initially published. A Pearson’s correlation coefficient test was applied to assess the correlation between the impact factor of the journal or years since publication or citation index and the number of citations for the top 100 cited articles included in the study. This was carried out using Statistical Package for Social Sciences (SPSS) Version 15.0. Descriptive statistics were presented as frequencies, medians and interquartile ranges or averages. p values <0.05 were considered statistically significant for all the comparisons.

## Results

### Citation count and publication year

The number of citations for the top 100 cited articles in paracetamol poisoning treatment ranged from 44 to 553 (Mitchell et al. 1974; Gazzard et al. 1975; Prescott et al. 1976, 1977, 1979, 1989; Rumack and Peterson 1978; Prescott 1981, 1983; Rumack et al. 1981; Forrest et al. 1982; Prescott and Critchley 1983; Black 1984; Mant et al. 1984; Rumack 1984, 1986; Seeff et al. 1986; Slattery et al. 1987; Lauterburg and Velez 1988; Smilkstein et al. 1988; Ziment 1988; Burgunder et al. 1989; Dawson et al. 1989; Riggs et al. 1989; Harrison et al. 1990; Murphy et al. 1990; Underhill et al. 1990; Flanagan and Meredith 1991; Harrison et al. 1991; Holdiness 1991; Keays et al. 1991; O’Grady et al. 1991; Penna and Buchanan 1991; Smilkstein et al. 1991; Bray et al. 1992; Jones and Vale 1993; Thomas 1993; Mutimer et al. 1994; Makin et al. 1995; Eguia and Materson 1997; Makin and Williams 1997; Bernal et al. 1998; Jones 1998; Kelly 1998; Mitchell et al. 1998; Perry and Shannon 1998; Anderson et al. 1999; Buckley et al. 1999a, b; Detry et al. 1999; McClain et al. 1999; Prescott 2000; Prince et al. 2000; Whyte et al. 2000; Woo et al. 2000; Ferner et al. 2001; Schmidt and Dalhoff 2001; Ward et al. 2001; Appelboam et al. 2002; Gyamlani and Parikh 2002; Rumack 2002; Schiodt et al. 2002; Schmidt et al. 2002; Wallace et al. 2002; James et al. 2003; Kao et al. 2003; Kozer et al. 2003; Daly et al. 2004; Lee 2004; Lynch and Robertson 2004; Benson et al. 2005; Kerr et al. 2005; Marzullo 2005; Prescott 2005; Sivilotti et al. 2005; Aitio 2006; Dart et al. 2006; Kanter 2006; Mahadevan et al. 2006; Nourjah et al. 2006; Atkuri et al. 2007; Larson 2007; Daly et al. 2008; Dodd et al. 2008; Fontana 2008; Heard 2008; Kortsalioudaki et al. 2008; Mazer and Perrone 2008; Pakravan et al. 2008; Waring et al. 2008c; Chun et al. 2009; James et al. 2009; Lee et al. 2009; Millea 2009; Sandilands and Bateman 2009; Yarema et al. 2009; Winnike et al. 2010; Ferner et al. 2011; Khandelwal et al. 2011; Antoine et al. 2013; Samuni et al. 2013) (see Table 1 for the top 100 cited publications in paracetamol poisoning treatment ranked in descending order of the number of citations). The median number of citations was 75 (interquartile range 56–137). These publications were published between 1974 and 2013. The average number of years since publication was 17.6 years, and 45 of the publications were from the 2000s. A significant, modest positive correlation was found between years since publication and the number of citations among the top 100 cited articles (r = 0.316; p = 0.001). The earliest article was written by Mitchell et al. (1974) almost 41 years ago in *Clinical Pharmacology and Therapeutics*, and the most recent were published about 2 years ago (2013) by Samuni et al. (2013) and Antoine et al. (2013) in *Biochimicaet Biophysica Acta*—*General Subjects* and *Hepatology* respectively.

**Table 1:** The top 100 cited publications in paracetamol poisoning treatment ranked in descending order of the number of citations

SCR	Authors	Title	Year	Source title	Cited by	Document type	IF^a^	Citation index	Country of corresponding author	Collaboration country
1st	Smilkstein et al. (1988)	Efficacy of oral *N*-acetylcysteine in the treatment of acetaminophen overdose. Analysis of the National Multicenter Study (1976 to 1985)	1988	*New England Journal of Medicine*	553	Article	55.873	20.5	USA	
2nd	James et al. (2003)	Acetaminophen-induced hepatotoxicity	2003	*Drug Metabolism and Disposition*	511	Review	3.252	42.6	USA	
3rd	Prescott et al. (1979)	Intravenous *N*-acetylcysteine: the treatment of choice for paracetamol poisoning	1979	*British Medical Journal*	419	Article	17.445	11.6	UK	
4th	Harrison et al. (1991)	Improvement by acetylcysteine of hemodynamics and oxygen transport in fulminant hepatic failure	1991	*New England Journal of Medicine*	340	Article	55.873	14.2	UK	
5th	Kelly (1998)	Clinical applications of *N*-acetylcysteine	1998	*Alternative Medicine Review*	297	Article	3.833	17.5	USA	
6th	Keays et al. (1991)	Intravenous acetylcysteine in paracetamol induced fulminant hepatic failure: a prospective controlled trial	1991	*British Medical Journal*	275	Article	17.445	11.5	UK	
7th	Mitchell et al. (1974)	Acetaminophen-induced hepatic injury: protective role of glutathione in man and rationale for therapy	1974	*Clinical Pharmacology and Therapeutics*	258	Article	7.903	6.3	USA	
8th	Seeff et al. (1986)	Acetaminophen hepatotoxicity in alcoholics. A therapeutic misadventure	1986	*Annals of Internal Medicine*	252	Review	17.810	8.7	USA	
9th	Lee (2004)	Acetaminophen and the US acute liver failure study group: lowering the risks of hepatic failure	2004	*Hepatology*	250	Review	11.055	22.7	USA	
10th	Rumack et al. (1981)	Acetaminophen overdose. 662 cases with evaluation of oral acetylcysteine treatment	1981	*Archives of Internal Medicine*	245	Article	17.333	7.2	USA	
11th	Harrison et al. (1990)	Improved outcome of paracetamol-induced fulminant hepatic failure by late administration of acetylcysteine	1990	*The Lancet*	239	Article	45.217	9.6	UK	
12th	Makin et al. (1995)	A 7-year experience of severe acetaminophen-induced hepatotoxicity (1987–1993)	1995	*Gastroenterology*	237	Article	16.716	11.9	UK	
13th	Forrest et al. (1982)	Clinical pharmacokinetics of paracetamol	1982	*Clinical Pharmacokinetics*	221	Review	5.053	6.7	UK	
14th	Prescott (1983)	Paracetamol overdosage. Pharmacological considerations and clinical management	1983	*Drugs*	219	Review	4.343	6.8	UK	
15th	Atkuri et al. (2007)	*N*-acetylcysteine—a safe antidote for cysteine/glutathione deficiency	2007	*Current Opinion in Pharmacology*	211	Review	4.595	26.4	USA	
16th	Lee et al. (2009)	Intravenous *N*-acetylcysteine improves transplant-free survival in early stage non-acetaminophen acute liver failure	2009	*Gastroenterology*	180	Article	16.716	30.0	USA	
17th	Black (1984)	Acetaminophen hepatotoxicity	1984	*Annual Review of Medicine*	178	Review	12.928	5.7	USA	
18th	Prescott et al. (1977)	Treatment of paracetamol (acetaminophen) poisoning with *n*-acetylcysteine	1977	*The Lancet*	175	Article	45.217	4.6	UK	
19th	Holdiness (1991)	Clinical pharmacokinetics of *N*-acetylcysteine	1991	*Clinical Pharmacokinetics*	172	Review	5.053	7.2	USA	
19th	Smilkstein et al. (1991)	Acetaminophen overdose: a 48-h intravenous *N*-acetylcysteine treatment protocol	1991	*Annals of Emergency Medicine*	172	Article	4.676	7.2	USA	
21st	Burgunder et al. (1989)	Effect of *N*-acetylcysteine on plasma cysteine and glutathione following paracetamol administration	1989	*European Journal of Clinical Pharmacology*	163	Article	2.966	6.3	Switzerland	
22nd	Lauterburg and Velez (1988)	Glutathione deficiency in alcoholics: risk factor for paracetamol hepatotoxicity	1988	*Gut*	159	Article	14.660	5.9	Switzerland	USA
23rd	Larson (2007)	Acetaminophen hepatotoxicity	2007	*Clinics in Liver Disease*	158	Review	3.660	19.8	USA	
24th	Rumack(2002)	Acetaminophen hepatotoxicity: the first 35 years	2002	*Journal of Toxicology*—*Clinical Toxicology*	148	Conference Paper	3.673	11.4	USA	
25th	Thomas (1993)	Paracetamol (acetaminophen) poisoning	1993	*Pharmacology and Therapeutics*	141	Article	9.723	6.4	UK	
26th	Bernal et al. (1998)	Use and outcome of liver transplantation in acetaminophen-induced acute liver failure	1998	*Hepatology*	134	Article	11.055	7.9	UK	
27th	Dodd et al. (2008)	*N*-acetylcysteine for antioxidant therapy: pharmacology and clinical utility	2008	*Expert Opinion on Biological Therapy*	133	Review	3.743	19.0	Australia	
28th	Heard (2008)	Acetylcysteine for acetaminophen poisoning	2008	*New England Journal of Medicine*	130	Article	55.873	18.6	USA	
29th	Chun et al. (2009)	Acetaminophen hepatotoxicity and acute liver failure	2009	*Journal of Clinical Gastroenterology*	121	Review	3.498	20.2	USA	
30th	Flanagan and Meredith (1991)	Use of *N*-acetylcysteine in clinical toxicology	1991	*The American Journal of Medicine*	117	Article	5.003	4.9	UK	
31st	Mant et al. (1984)	Adverse reactions to acetylcysteine and effects of overdose	1984	*British Medical Journal*	115	Article	17.445	3.7	UK	
32nd	Prescott et al. (1989)	The disposition and kinetics of intravenous *N*-acetylcysteine in patients with paracetamol overdosage	1989	*European Journal of Clinical Pharmacology*	114	Article	2.966	4.4	UK	
33rd	Daly et al. (2008)	Guidelines for the management of paracetamol poisoning in Australia and New Zealand—explanation and elaboration	2008	*Medical Journal of Australia*	109	Article	4.089	15.6	Australia	New Zealand
34th	Nourjah et al. (2006)	Estimates of acetaminophen (paracetamol)-associated overdoses in the United States	2006	*Pharmacoepidemiology and Drug Safety*	103	Article	2.939	11.4	USA	
34th	Schmidt et al. (2002)	Acute versus chronic alcohol consumption in acetaminophen-induced hepatotoxicity	2002	*Hepatology*	103	Article	11.055	7.9	Denmark	
36th	Rumack and Peterson (1978)	Acetaminophen overdose: incidence, diagnosis, and management in 416 patients	1978	*Pediatrics*	102	Article	5.473	2.8	USA	
37th	Mazer and Perrone(2008)	Acetaminophen-induced nephrotoxicity: pathophysiology, clinical manifestations, and management.	2008	*Journal of Medical Toxicology*: *Official Journal of the American College of Medical Toxicology*	96	Review	NA	13.7	USA	
38th	Jones (1998)	Mechanism of action and value of *N*-acetylcysteine in the treatment of early and late acetaminophen poisoning: a critical review	1998	*Journal of Toxicology*—*Clinical Toxicology*	94	Review	3.673	5.5	UK	
39th	Ward et al. (2001)	Acetaminophen toxicity in children	2001	*Pediatrics*	93	Review	5.473	6.6	USA	Canada
39th	Prescott et al. (1976)	Cysteamine, methionine, and penicillamine in the treatment of paracetamol poisoning	1976	*The Lancet*	93	Article	45.217	2.4	UK	
41st	Penna and Buchanan (1991)	Paracetamol poisoning in children and hepatotoxicity	1991	*British Journal of Clinical Pharmacology*	92	Review	3.878	3.8	Australia	
42nd	Prescott and Critchley (1983)	The treatment of acetaminophen poisoning	1983	*Annual Review of Pharmacology and Toxicology*	90	Review	18.365	2.8	UK	
43rd	Prescott (2000)	Paracetamol: past, present, and future	2000	*American Journal of Therapeutics*	89	Article	1.129	5.9	UK	
44th	Buckley et al. (1999b)	Oral or intravenous *N*-acetylcysteine: which is the treatment of choice for acetaminophen (paracetamol) poisoning?	1999	*Journal of Toxicology*—*Clinical Toxicology*	88	Article	3.673	5.5	Australia	
45th	Antoine et al. (2013)	Mechanistic biomarkers provide early and sensitive detection of acetaminophen-induced acute liver injury at first presentation to hospital	2013	*Hepatology*	84	Article	11.055	42.0	UK	Switzerland
46th	Mitchell et al. (1998)	Earlier identification of patients at risk from acetaminophen-induced acute liver failure	1998	*Critical Care Medicine*	83	Article	6.312	4.9	UK	
46th	Slattery et al. (1987)	Dose-dependent pharmacokinetics of acetaminophen: evidence of glutathione depletion in humans	1987	*Clinical Pharmacology and Therapeutics*	83	Article	7.903	3.0	USA	
48th	Millea (2009)	*N*-acetylcysteine: multiple clinical applications	2009	*American Family Physician*	79	Review	2.175	13.2	USA	
49th	Samuni et al. (2013)	The chemistry and biological activities of *N*-acetylcysteine	2013	*Biochimica et BiophysicaActa*—*General Subjects*	76	Review	4.381	38.0	Australia	Israel
50th	Winnike et al. (2010)	Use of pharmaco-metabonomics for early prediction of acetaminophen-induced hepatotoxicity in humans	2010	*Clinical Pharmacology and Therapeutics*	75	Article	7.903	15.0	USA	
50th	Rumack (1984)	Acetaminophen overdose in young children: treatment and effects of alcohol and other additional ingestants in 417 cases	1984	*American Journal of Diseases of Children*	75	Article	Stop	2.4	USA	
52nd	Kerr et al. (2005)	The Australasian clinical toxicology investigators collaboration randomized trial of different loading infusion rates of *N*-acetylcysteine	2005	*Annals of Emergency Medicine*	74	Article	4.676	7.4	Australia	
53rd	Aitio(2006)	*N*-acetylcysteine—passe-partout or much ado about nothing?	2006	*British Journal of Clinical Pharmacology*	73	Review	3.878	8.1	Switzerland	
53rd	Detry et al. (1999)	Clinical use of a bioartificial liver in the treatment of acetaminophen-induced fulminant hepatic failure	1999	*American Surgeon*	73	Article	0.818	4.6	USA	
55th	Dart et al. (2006)	Acetaminophen poisoning: an evidence-based consensus guideline for out-of-hospital management	2006	*Clinical Toxicology*	72	Review	3.673	8.0	USA	
56th	Eguia and Materson (1997)	Acetaminophen-related acute renal failure without fulminant liver failure	1997	*Pharmacotherapy*	71	Article	2.662	3.9	USA	
56th	Dawson et al. (1989)	Adverse reactions to *N*-acetylcysteine during treatment for paracetamol poisoning	1989	*Medical Journal of Australia*	71	Article	4.089	2.7	Australia	
58th	Schmidt and Dalhoff (2001)	Risk factors in the development of adverse reactions to *N*-acetylcysteine in patients with paracetamol poisoning	2001	*British Journal of Clinical Pharmacology*	70	Article	3.878	5.0	DenmarK	
59th	Woo et al. (2000)	Shorter duration of oral *N*-acetylcysteine therapy for acute acetaminophen overdose	2000	*Annals of Emergency Medicine*	68	Article	4.676	4.5	USA	
60th	Buckley et al. (1999a)	Activated charcoal reduces the need for *N*-acetylcysteine treatment after acetaminophen (paracetamol) overdose	1999	*Journal of Toxicology*—*Clinical Toxicology*	67	Article	3.673	4.2	Australia	
61st	Ziment (1988)	Acetylcysteine: a drug that is much more than a mucokinetic	1988	*Biomedicine and Pharmacotherapy*	66	Review	2.023	2.4	USA	
62nd	James et al. (2009)	Pharmacokinetics of acetaminophen-protein adducts in adults with acetaminophen overdose and acute liver failure	2009	*Drug Metabolism and Disposition*	64	Article	3.252	10.7	USA	
62nd	Fontana (2008)	Acute liver failure including acetaminophen overdose	2008	*Medical Clinics of North America*	64	Review	2.607	9.1	USA	
64th	Benson et al. (2005)	The therapeutic use of acetaminophen in patients with liver disease	2005	*American Journal of Therapeutics*	63	Review	1.129	6.3	USA	
64th	Bray et al. (1992)	Long-term anticonvulsant therapy worsens outcome in paracetamol-induced fulminant hepatic failure	1992	*Human and Experimental Toxicology*	63	Article	1.747	2.7	Denmark	
66th	Prescott (2005)	Oral or intravenous *N*-acetylcysteine for acetaminophen poisoning?	2005	*Annals of Emergency Medicine*	61	Editorial	4.676	6.1	UK	
66th	Perry and Shannon (1998)	Efficacy of oral versus intravenous *N*-acetylcysteine in acetaminophen overdose: results of an open-label, clinical trial	1998	*Journal of Pediatrics*	61	Article	3.790	3.6	USA	
68th	Sandilands and Bateman (2009)	Adverse reactions associated with acetylcysteine Adverse reactions associated with acetylcysteine	2009	*Clinical Toxicology*	60	Review	3.673	10.0	UK	
68th	Ferner et al. (2001)	Random and systematic medication errors in routine clinical practice: a multicentre study of infusions, using acetylcysteine as an example	2001	*British Journal of Clinical Pharmacology*	60	Article	3.878	4.3	UK	
68th	O’Grady et al. (1991)	Liver transplantation after paracetamol overdose	1991	*British Medical Journal*	60	Article	17.445	2.5	UK	
71st	Kanter (2006)	Comparison of oral and i.v. acetylcysteine in the treatment of acetaminophen poisoning	2006	*American Journal of Health*-*System Pharmacy*	59	Review	1.882	6.6	USA	
72nd	Appelboam et al. (2002)	Fatal anaphylactoid reaction to *N*-acetylcysteine: caution in patients with asthma	2002	*Emergency Medicine Journal*	58	Article	1.843	4.5	UK	
72nd	Gazzard et al. (1975)	Early changes in coagulation following a paracetamol overdose and a controlled trial of fresh frozen plasma therapy	1975	*Gut*	58	Article	14.660	1.5	UK	
74th	Kortsalioudaki et al. (2008)	Safety and efficacy of *N*-acetylcysteine in children with non-acetaminophen-induced acute liver failure	2008	*Liver Transplantation*	57	Article	4.241	8.1	UK	
74th	Mutimer et al. (1994)	Serious paracetamol poisoning and the results of liver transplantation	1994	*Gut*	57	Article	14.660	2.7	UK	
74th	Jones and Vale (1993)	Paracetamol poisoning and the kidney	1993	*Journal of Clinical Pharmacy and Therapeutics*	57	Review	1.668	2.6	UK	
77th	Kao et al. (2003)	What Is the rate of adverse events after oral *N*-acetylcysteine Administered by the intravenous route to patients with suspected acetaminophen poisoning?	2003	*Annals of Emergency Medicine*	56	Review	4.676	4.7	US	
77th	Wallace et al. (2002)	Paracetamol overdose: an evidence based flowchart to guide management	2002	*Emergency Medicine Journal*	56	Article	1.843	4.3	UK	
79th	McClain et al. (1999)	Acetaminophen hepatotoxicity: an update	1999	*Current gastroenterology reports*	55	Review	NA	3.4	USA	
79th	Makin and Williams (1997)	Acetaminophen-induced hepatotoxicity: predisposing factors and treatments	1997	*Advances in internal medicine*	55	Review	NA	3.1	UK	
79th	Prescott (1981)	Treatment of severe acetaminophen poisoning with intravenous acetylcysteine	1981	*Archives of Internal Medicine*	55	Article	17.333	1.6	UK	
82nd	Murphy et al. (1990)	Severe acetaminophen toxicity in a patient receiving isoniazid	1990	*Annals of Internal Medicine*	53	Article	17.810	2.1	USA	
83rd	Daly et al. (2004)	Prospective evaluation of repeated supratherapeutic acetaminophen (paracetamol) ingestion	2004	*Annals of Emergency Medicine*	52	Article	4.676	4.7	USA	
83rd	Kozer et al. (2003)	Glutathione, glutathione-dependent enzymes and antioxidant status in erythrocytes from children treated with high-dose paracetamol	2003	*British Journal of Clinical Pharmacology*	52	Article	3.878	4.3	Israel	
83rd	Prince et al. (2000)	Reduction in incidence of severe paracetamol poisoning	2000	*The Lancet*	52	Note	45.217	3.5	UK	
83rd	Riggs et al. (1989)	Acute acetaminophen overdose during pregnancy	1989	*Obstetrics and Gynecology*	52	Article	5.175	2.0	USA	
87th	Khandelwal et al. (2011)	Unrecognized acetaminophen toxicity as a cause of indeterminate acute liver failure	2011	*Hepatology*	51	Article	11.055	12.8	USA	
87th	Yarema et al. (2009)	Comparison of the 20-h intravenous and 72-h oral acetylcysteine protocols for the treatment of acute acetaminophen poisoning	2009	*Annals of Emergency Medicine*	51	Article	4.676	8.5	Canada	USA
87th	Lynch and Robertson (2004)	Anaphylactoid reactions to intravenous *N*-acetylcysteine: a prospective case controlled study	2004	*Accident and Emergency Nursing* (*Continue as International Emergency Nursing*)	51	Article	0.703	4.6	UK	
90th	Ferner et al. (2011)	Management of paracetamol poisoning	2011	*British Medical Journal*	50	Review	17.445	12.5	UK	
90th	Mahadevan et al. (2006)	Paracetamol induced hepatotoxicity	2006	*Archives of Disease in Childhood*	50	Review	2.899	5.6	UK	
90th	Gyamlani and Parikh (2002)	Acetaminophen toxicity: suicidal versus accidental	2002	*Critical Care*	50	Article	4.476	3.8	USA	
90th	Underhill et al. (1990)	A comparison of the efficacy of gastric lavage, ipecacuanha and activated charcoal in the emergency management of paracetamol overdose	1990	*Archives of Emergency Medicine* (*Continue as Emergency Medicine Journal*)	50	Article	1.843	2.0	UK	
94th	Waring et al. (2008c)	Lower incidence of anaphylactoid reactions to *N*-acetylcysteine in patients with high acetaminophen concentrations after overdose	2008	*Clinical Toxicology*	49	Article	3.673	7.0	UK	
94th	Whyte et al. (2000)	Acetaminophen causes an increased international normalized ratio by reducing functional factor VII	2000	*Therapeutic Drug Monitoring*	49	Article	2.376	3.3	Australia	
94th	Anderson et al. (1999)	Predicting concentrations in children presenting with acetaminophen overdose	1999	*Journal of Pediatrics*	49	Article	3.790	3.1	New Zealand	
97th	Rumack (1986)	Acetaminophen overdose in children and adolescents	1986	*Pediatric Clinics of North America*	48	Article	2.120	1.7	USA	
98th	Marzullo (2005)	An update of *N*-acetylcysteine treatment for acute acetaminophen toxicity in children	2005	*Current Opinion in Pediatrics*	47	Review	2.528	4.7	USA	
99th	Sivilotti et al. (2005)	A risk quantification instrument for acute acetaminophen overdose patients treated with *n*-acetylcysteine	2005	*Annals of Emergency Medicine*	44	Article	4.676	4.6	Canada	USA
99th	Schiodt et al. (2002)	Influence of acute and chronic alcohol intake on the clinical course and outcome in acetaminophen overdose	2002	*Alimentary Pharmacology and Therapeutics*	44	Article	5.727	3.5	Denmark	USA
99th	Pakravan et al. (2008)	Risk factors and mechanisms of anaphylactoid reactions to acetylcysteine in acetaminophen overdose	2008	*Clinical Toxicology*	44	Article	3.673	6.3	UK	

Equal articles have the same ranking number, and then a gap is left in the ranking numbers

*SCR* standard competition ranking

^a^The impact factor was reported according to the Institute for Scientific Information (ISI) journal citation reports (JCR) 2014

### Average number of citations per year

Average number of citations per year for the top 100 cited articles ranged from 1.5 to 42.6 with a mean of 8.5 citations per year and a median of 5.9 (interquartile range 3.75–10.35). Table 2 ranks the top 10 publications based on the highest average number of citations per year. Nine of the articles in the list were published after year 2000. The top three articles based on average number of citations per year were the followings: “Acetaminophen-induced hepatotoxicity” with 42.6 average number of citations per year, “Mechanistic biomarkers provide early and sensitive detection of acetaminophen-induced acute liver injury at first presentation to hospital” with 42.0 average number of citations per year and “The chemistry and biological activities of *N*-acetylcysteine” with 38.6 average number of citations per year. Interestingly, the total number of citations was significantly correlated with citation index (r = 0.485, p < 0.001).

**Table 2:** Ranking the top 10 articles in paracetamol poisoning treatment based on average citations per year

SCR	Authors	Title	Year	Source title	Cited by	Document type	IF^a^	Citation index	Country of corresponding author	Collaboration country
1st	James et al. (2003)	Acetaminophen-induced hepatotoxicity	2003	*Drug Metabolism and Disposition*	511	Review	3.252	42.6	USA	
2nd	Antoine et al. (2013)	Mechanistic biomarkers provide early and sensitive detection of acetaminophen-induced acute liver injury at first presentation to hospital	2013	*Hepatology*	84	Article	11.055	42.0	UK	Switzerland
3rd	Samuni et al. (2013)	The chemistry and biological activities of *N*-acetylcysteine	2013	*Biochimica et BiophysicaActa* - *General Subjects*	76	Review	4.381	38.0	Australia	Israel
4th	Lee et al. (2009)	Intravenous *N*-acetylcysteine improves transplant-free survival in early stage non-acetaminophen acute liver failure	2009	*Gastroenterology*	180	Article	16.716	30.0	USA	
5th	Atkuri et al. (2007)	*N*-acetylcysteine—a safe antidote for cysteine/glutathione deficiency	2007	*Current Opinion in Pharmacology*	211	Review	4.595	26.4	USA	
6th	Lee (2004)	Acetaminophen and the US acute liver failure study group: lowering the risks of hepatic failure	2004	*Hepatology*	250	Review	11.055	22.7	USA	
7th	Smilkstein et al. (1988)	Efficacy of oral *N*-acetylcysteine in the treatment of acetaminophen overdose. Analysis of the National Multicenter Study (1976–1985)	1988	*New England Journal of Medicine*	553	Article	55.873	20.5	USA	
8th	Chun et al. (2009)	Acetaminophen hepatotoxicity and acute liver failure	2009	*Journal of Clinical Gastroenterology*	121	Review	3.498	20.2	USA	
9th	Larson (2007)	Acetaminophen hepatotoxicity	2007	*Clinics in Liver Disease*	158	Review	3.660	19.8	USA	
10th	Dodd et al. (2008)	*N*-acetylcysteine for antioxidant therapy: pharmacology and clinical utility	2008	*Expert Opinion on Biological Therapy*	133	Review	3.743	19.0	Australia	

*SCR* standard competition ranking

^a^The impact factor was reported according to the Institute for Scientific Information (ISI) journal citation reports (JCR) 2014

### Authorship

The total number of authors for the top 100 cited articles was 419, for an average of 4.15 authors per paper. Authors per paper ranged from 1 to 23, and 20 articles were written by one author. Table 3 ranks the top 10 most prolific authors, who have published at least four publications among the 100 most cited publications.

**Table 3:** The top 10 ranking of prolific authors who published most frequently cited publications, with their affiliations

SCR	Author	No. of publications	Affiliation
1st	Prescott, L.	10	University of Edinburgh, Edinburgh, Scotland, UK
1st	Williams, R.	10	Foundation for Liver Research, Institute of Hepatology, London, UK
3rd	Rumack, B. H.	9	Department of Emergency Medicine, University of Colorado School of Medicine, Aurora, CO, USA
4th	Wendon, J.	6	Institute of Liver Studies at King’s College School of Medicine at King’s College Hospital, London, UK
5th	Bateman, D. N.	5	University of Edinburgh, Edinburgh, UK
5th	Buckley, N.	5	Department of Pharmacology, School of Medical Sciences, University of Sydney, Sydney, New South Wales, Australia
5th	Dawson, A.	5	NSW Poisons Information Center, Westmead Children’s Hospital Sydney, Australia
5th	Lee, W. M.	5	Division of Digestive and Liver Diseases, University of Texas Southwestern Medical Center at Dallas, 5959 Harry Hines Boulevard, Suite 420, Dallas, TX 75390-8887, USA
9th	Alexander, G.J.M.	4	Institute of Liver Studies, King’s College School of Medicine and Dentistry, London, UK
9th	Harrison, P.M.	4	Institute of Liver Studies, King’s College Hospital, Denmark Hill, London, UK
9th	Whyte, I.M.	4	Calvary Mater Newcastle, Newcastle, NSW, Australia

*SCR* standard competition ranking

### Journals

A total of 55 journals published these top 100 cited articles. Nine documents were published in *Clinical Toxicology*, whereas eight documents were published in *Annals of Emergency Medicine* and five documents each were published in *British Journal of Clinical Pharmacology*, *Hepatology* and *British Medical Journal*. The impact factor for journals containing the top 100 cited paracetamol poisoning treatment articles ranged from 0.703 to 55.873. Twenty-eight documents were published in the ten journals with an IF >10. Only three journals for the top 100 cited articles were without IF. A significant, modest positive correlation was found between the journal impact factor and the number of citations among the top 100 cited articles (r = 0.426; p < 0.001).

### Origins and publication type

In relation to the origin of the research publications for the highly cited articles, they were from eight countries. The USA had the largest number of articles with 47 articles. The UK and Australia published 38 and nine articles respectively, whereas Denmark, Switzerland, Canada, New Zealand and Israel published four, four, three, two and two articles respectively. In the terms of “collaboration with other countries”, we found that eight articles were published and co-authored by researchers from multiple countries. Sixty-six articles were original articles, 32 articles were review articles and three were other types of publications, including conference papers, editorials and notes.

## Discussion

The present study was designed to rank and characterize the top 100 cited publications in the field of paracetamol poisoning treatment. The most obvious finding to emerge from this study is that results of this study may explain how developments in this area of clinical toxicology have progressed over time. It becomes obvious which key publications and authors have made exceptional contributions that have played an integral role in shaping the guidelines related to the treatment of paracetamol. Furthermore, the results of this study enhance our understanding about the leading publications that have contributed to the development of this field of toxicology.

Common treatment guidelines for paracetamol poisoning that are currently in use include Australian and New Zealand guideline by Daly et al. (2008), USA guideline by Dart et al. (2006) and UK guideline by Wallace et al. (2002). These guidelines ranked 33rd, 55th and 77th respectively. Furthermore, these common guidelines were based on several articles in the top 100 cited publications such as Smilkstein et al. (1988), Prescott et al. (1979), Keays et al. (1991), Prescott et al. (1977), Rumack (2002), and Smilkstein et al. (1991).

The top 100 cited studies in our study were published from 1974 to 2013 with a citation range of 44–553 times since publication. Compared to citations in other medical fields, this occupies a low position; in tuberculosis, the number of citations for top-cited studies ranged from 366 to 4443 (Chen et al. 2015), compared to 582–7248 for hypertension (Oh and Galis 2014). The difference in the total number of citations in each area reveals the number of researchers working in each area (Chen et al. 2015). It is also well known that editors for the main journals with high IFs consider articles with a high citation rate to sustain the IF progress of their journals (Zyoud et al. 2014a, b, 2015a, b, d). Furthermore, since the toxicology field is considered as a very narrow field with a very small readership and number of researchers, it should not be surprising for toxicology topics or toxicology journals to have a small numbers of citations (Bird 2008; Zyoud et al. 2014b). Our results confirm that there was a modest relationship between the number of years elapsed since the time of publication and the number of citations. These results support previous findings which reported that older articles attained more citations because their citable period was longer (Loonen et al. 2008; Lefaivre et al. 2011; Aminian et al. 2014; Joyce et al. 2014; Lee et al. 2015). The annual total number of citations for any article fluctuates with time and for some articles the total annual number of citations might decrease with time while for other articles, the total annual number of citations might increase or remain steady with time. In our study, the low correlation between the total citations and citation index seems to be related to papers that have many citations when they are first published but then drop off in later years because researchers might be tended to preferentially cite the most recent studies (Azer and Azer 2016). Our study showed that the average number of citations per year for articles published after year 2000 was higher than those published before 2000. This finding may be due to the tendency of authors to cite recent papers which is a common practice among authors (Van Noorden et al. 2014).

The most influential article in paracetamol poisoning treatment was conducted by Smilkstein and experts from the USA (Smilkstein et al. 1988) and was published in 1988 in *New England Journal of Medicine*. This article described the final outcomes of 2540 patients with paracetamol overdose treated with a loading dose of 140 mg per kg of oral NAC followed by 70 mg per kg given every 4 h for an additional 17 doses. It was concluded that NAC treatment should be started within 8 h of an paracetamol overdose (Smilkstein et al. 1988). In contrast to earlier findings, recently, in the last decade, described novel NAC infusion regimens offer different rates of intravenous NAC administration in both the loading and maintenance doses, and this is associated with a lower rate of the occurrence of adverse effects (Pakravan et al. 2008; Waring et al. 2008c). These studies were in the list of influential papers in the top 100 cited articles.

The first paper for paracetamol poisoning treatment was cited only 25 times and was published by Maclean et al. (1968) in 1968 in *The Lancet*. This article recommended immediate gastric lavage, intravenous hydrocortisone, forced diuresis and antihistamine for paracetamol poisoning treatment. This article achieved a low rate of citation to be listed in the top 100 cited publications in the field of paracetamol poisoning treatment because it was based on treatment regimens, which limited recommendations for the treatment of such cases. The two pioneering publications in the field of general paracetamol poisoning were conducted by Mitchell et al. (1973a, b). They explained the mechanism of paracetamol hepatotoxicity, were published in *Journal of Pharmacology and Experimental Therapeutics* and are considered as remarkable papers in paracetamol poisoning (Rumack and Bateman 2012). These two publications achieved higher citations in the field of general paracetamol poisoning than in the field of paracetamol poisoning treatment, 1052 and 904 citations each respectively (data not shown), but did not appear in our list because they involved animal research. What is surprising is that this group was in our list and got an advanced position (i.e. the seventh most frequently cited paper) by providing a rationale for therapy in humans by indicating that cysteamine could prevent hepatotoxicity (Mitchell et al. 1974). Prescott et al. (1974) reported the successful treatment of patients with severe paracetamol overdose with cysteamine, but this article was not among the top 100 cited articles. This might be due to the adverse effects in patients making it a less than ideal antidote and of low interest to researchers. Surprisingly, Prescott et al. (1979) were found to be in the list of the top 100 cited articles for a different article, which achieved the third position in the list. They reported that intravenous NAC was more effective in the treatment of paracetamol poisoning than cysteamine and methionine and was markedly free from adverse effects (Prescott et al. 1979). Furthermore, it is somewhat surprising that Prescott and his colleagues in Edinburgh published a series of influential papers in the top 100 cited articles (Prescott et al. 1976, 1977, 1989; Prescott 1983, 2000, 2005; Prescott and Critchley 1983).

One of the pioneering article in the field of general paracetamol poisoning was written by Rumack and Matthew (1975). This article is considered as one of the highly cited articles in the field of general paracetamol poisoning, rather than in the field of paracetamol poisoning treatment. It achieved 303 citations (data not shown) but did not appear in our list. Contrary to expectations, several studies (Schiodt et al. 1997; Larson et al. 2005) were dropped from the list of the top 100 cited articles in the field of paracetamol poisoning treatment because these studies did not mention paracetamol poisoning related treatment terms in their titles or abstract. The most obvious finding to emerge from our study is that Rumack published a series of influential papers in the top 100 cited articles (Rumack and Peterson 1978; Rumack et al. 1981; Rumack 1984, 1986, 2002).

In the present study, 28 documents were published in the ten journals with an IF >10, including *New England Journal of Medicine*, *The Lancet*, *Annual Review of Pharmacology and Toxicology*, *Annals of Internal Medicine*, *British Medical Journal*, *Archives of Internal Medicine*, *Gastroenterology*, *Gut*, *Annual Review of Medicine* and *Hepatology*. Our results confirm the modes relationship between the journal impact factor and the number of citations among the top 100 cited articles. These results seem to be consistent with other research, which found that the most cited articles in the field of tuberculosis are often published in journals that top the impact factor list (Chen et al. 2015). The results of our study show that more than half of the publications originated from the USA, followed by the UK. These results match those observed in earlier studies (Loonen et al. 2008; Joyce et al. 2014; Oh and Galis 2014; Chen et al. 2015; Dolan et al. 2015). Research activity in these countries is most likely due to their economic strength (Li et al. 2013; Yun et al. 2015), or numerous large poison centers (Forrester 2016), or the number of researchers or general research activity in this scientific field (Zyoud et al. 2015a, d), or number of poisoning incidents in these countries (Bateman 2014; Mowry et al. 2015).

There are several limitations of this study. First, our analysis to choose publications with a primary focus on paracetamol poisoning treatment research likely excluded articles that had otherwise notably influenced thinking in the field, including some of the highly cited articles that refer to investigations of paracetamol poisoning in animals or in vitro. Second, it was based on the Scopus database alone; Scopus does not index all journals, and we may have missed journals that figure in other databases such as Google Scholar. Third, articles published in recent years had less of a chance to be among the top 100 cited articles because less time has elapsed since the date of publication to allow citation. Another limitation is that some publications did not mention paracetamol poisoning related treatment terms in their titles or abstract, so it is possible that not all publications about paracetamol were considered. The present study design means that we are unable to include some clinicians that have influenced the treatment of paracetamol poisoning through other means, including unpublished research work, presentations at scientific meetings, and pioneering clinical practice. For example, many influential toxicologists, such as Matthew H., Jaeschke H., Piperno E. and Khairallah E.A. (Rumack 2002; Proudfoot and Prescott 2009; Rumack and Bateman 2012), were not identified in our study.

In conclusion, we carried out a bibliometric analysis of the most cited publications focused on paracetamol poisoning research, revealing a number of characteristics related to these influential publications, including the country of origin, type of study, journal and authorship. This study is the first bibliometric assessment of the top 100 cited articles in toxicology literature. The most influential report in paracetamol poisoning treatment research appears to be conducted by Smilkstein et al. from the USA and was published by in 1988 in *New England Journal of Medicine*. Interest in paracetamol poisoning as a serious clinical problem continues to grow. Research published in high-impact journals and from high income countries is most likely to be cited in published paracetamol research.

## Abbreviations

NAC: *N*-acetylcysteine
SPSS: Statistical Package for Social Sciences
ISI: Institute for Scientific Information
JCR: Journal Citation Reports
IFs: impact factors
SCR: standard competition ranking
